# A Discrete Choice Experiment to Assess Cat Owners’ Preferences for Topical Antiparasitics and the Comparative Ease of Use of a Combined Selamectin and Sarolaner Formulation: An International Survey

**DOI:** 10.3390/ani15131985

**Published:** 2025-07-06

**Authors:** Constantina N. Tsokana, George Valiakos, Kennedy Mwacalimba, Danielle Riley, Ashley Enstone, Robin Wyn, Tom Metcalf, Emily Melchior, Eleni Pavlidou, Andrea Wright

**Affiliations:** 1 Laboratory of Parasitology and Parasitic Diseases, School of Veterinary Medicine, Faculty of Health Sciences, Aristotle University of Thessaloniki, 54124 Thessaloniki, Greece; ctsokana@vet.auth.gr; 2 Laboratory of Microbiology and Parasitology, Faculty of Veterinary Science, University of Thessaly, 43100 Karditsa, Greece; georgevaliakos@uth.gr; 3 In-Market Clinical Studies and Outcomes Research, Global Medical Affairs, Zoetis, Parsippany, NJ 07054, USA; kennedy.mwacalimba@zoetis.com (K.M.); emily.melchior@zoetis.com (E.M.); 4 Adelphi Values, PROVE, Adelphi Mill, Bollington SK10 5JB, Cheshire, UK; danielle.riley@adelphivalues.com (D.R.); ashley.enstone@adelphivalues.com (A.E.); robin.wyn@adelphivalues.com (R.W.); tom.metcalf@bath.edu (T.M.); 5 Asclepius One Health Platform, 10671 Athens, Greece; info@asclepiusoh.com

**Keywords:** antiparasitics, cats, discrete choice experiment, ease of use, pet owners, physical properties, preferences

## Abstract

Cat owners are involved in their cats’ healthcare, including the prevention of parasitic diseases. However, a comprehensive understanding of their preferences for feline antiparasitics is lacking. This study addresses this gap through a multifaceted methodology comprising three phases. In Phase 1, the physical properties and usability aspects of seven topical antiparasitic formulations were assessed. Within Phase 2, an ease-of-use study was conducted to evaluate the cat owners’ application experience with deidentified products representing three topical antiparasitics. Phase 3 included the identification and validation of product attributes most valued by pet owners through interviews with cat owners and veterinary experts. The product attributes identified informed the subsequent quantitative discrete choice experiment (DCE), which involved 1040 cat owners from different countries (Australia/New Zealand, Canada, Greece/Spain, and the UK) and aimed to analyze their preferences based on choices among product profiles mirroring four topical antiparasitics: selamectin–sarolaner, moxidectin–fluralaner, moxidectin–imidacloprid, and eprinomectin–esafoxolaner–praziquantel. Phase 1 showed that the selamectin–sarolaner formulation exhibits minimal odor, less stickiness, and less drying time. The ease-of-use study (Phase 2) showed that the blinded product representing the selamectin–sarolaner formulation was characterized by seamless application, rapid dispensing, and a sense of control during application. The quantitative DCE study (Phase 3) indicated a preference for the product profile mirroring the selamectin–sarolaner formulation among a global sample of cat owners. Demographic characteristics such as gender, age, and insurance status influenced their preferences. Key predictors for preferring the selamectin–sarolaner formulation over at least one comparator treatment included the ability to confirm successful administration, age restrictions, ease of application, and the time before the cat could sit on furniture following administration. These findings suggest that cat owners prioritize ease of use, safety, and overall user experience, providing valuable guidance for veterinary practitioners to make informed treatment recommendations.

## Simple Summary

Cat owners are involved in their cats’ healthcare, including the prevention of parasitic diseases. However, we do not fully understand the factors that influence their preferences when choosing antiparasitic treatments. This study, comprising three phases, aimed to fill that gap. In Phase 1, several features and usability aspects of seven topical antiparasitics were assessed. In Phase 2, cat owners evaluated their application experience with blinded products representing three topical antiparasitics. Phase 3 included interviews with pet owners and veterinary experts to identify and validate the product attributes most valued by pet owners when choosing topical antiparasitics. These product attributes were then used in an international survey involving 1040 cat owners from different countries (Australia/New Zealand, Canada, Greece/Spain, and the UK) to understand their preferences among product profiles mirroring four topical antiparasitics. Phase 1 showed that the selamectin–sarolaner formulation had minimal smell, was not sticky, and dried quickly. In Phase 2, participants characterized the blinded product representing the selamectin–sarolaner formulation as having a seamless application, rapid dispensing, and a sense of control during application. In Phase 3, a global sample of cat owners showed preference for the product profile mirroring the selamectin–sarolaner treatment. Factors like age, gender, and insurance influenced their choices. The ability to confirm successful administration, age restrictions, ease of application, and the time before the cat could sit on furniture following application significantly influenced cat owners’ preference for the product profile mirroring the selamectin–sarolaner formulation over at least one comparator treatment. These findings indicate that cat owners prioritize ease of use and safety, which can help veterinarians make better recommendations for pet treatments, leading to healthier cats.

## 1. Introduction

The domestication of cats represents a unique human–animal relationship in history; evidence dates back 9500 years to a human and cat shared burial in Cyprus [1]. Unlike other animals, cats were not primarily domesticated for practical purposes such as food. Instead, their natural ability to control pests, like mice and snakes, contributed to their integration into human settlements [2]. This mutually beneficial relationship gradually evolved into a more complex social bond, where the cats’ inherent hunting prowess was complemented by their appealing physical features and independent yet affectionate nature [1,3]. As a result, cats have become companion animals that maintain much of their ancestral behavior while forming deep emotional bonds with humans—a partnership that continues to evolve [3].

Today, cats are popular pets globally. Although they retain a significant degree of independence, as evidenced by the large population of feral cats that thrive without regular human care [4], the human–cat bond has grown stronger [5].

However, this close relationship also brings health considerations, particularly regarding parasitic infections. Cats can harbor both endoparasites and ectoparasites, with varying prevalence rates among countries and cat populations [6,7]. The prevalence of ectoparasites and endoparasites in owned cats in nine European countries was 29.6% and 35.1%, respectively; *Otodectes cynotis* (17.4%) and fleas (15.5%) were the most frequently identified ectoparasites, while *Toxocara cati* (19.7%) was the most prevalent nematode. Notably, 14% of the cats were co-infested with endoparasites and ectoparasites, demonstrating the need for effective treatment against both [6]. Similar findings come from other continents, with the following reported pooled prevalences for *T. cati*: 27.9% in Asia, 21.4% in Africa, and 18.5% in North America [8]. Other studies reached similar conclusions and highlighted the need for year-round treatment in cats [9,10] and a higher frequency of parasitic infections in stray or feral cats [11].

The management of parasites has evolved significantly; endo- and ectoparasiticides and endectocides are used prophylactically or therapeutically. Current treatment options include, among others, macrocyclic lactones, pyrethroids, organophosphates, and the newer class of isoxazolines [12]. According to European Scientific Counsel Companion Animal Parasites (ESCCAP) guidelines, cats living outdoors should receive endoparasitic treatments at least four times annually, while cats living indoors require treatment one to two times yearly. As the infestation risk increases based on the lifestyle of the cats and the local parasite prevalence, they should receive year-round protection against ticks, fleas, and heartworm disease [13,14,15]. The increasing cat population in urban areas worldwide [16] necessitates continued innovation in parasite control strategies. Developing effective and more convenient treatment options with broad coverage of endo- and ectoparasites in one application remains essential for ensuring feline health and the safety of the human–animal bond [13].

Veterinary professionals play a crucial role in communicating cat and human health risks due to parasites, and they are also responsible for recommending appropriate treatments [17,18]. Antiparasitics are available worldwide with a veterinary prescription. However, regulations on their prescription vary across countries [19]. In some regions, there is no prescription requirement, placing the responsibility of choosing antiparasitic treatments on the pet owners. However, consulting a veterinarian is recommended to ensure the appropriateness of the treatment. Moreover, despite the availability of antiparasitics, adherence to recommended protocols is challenging, and the actual compliance rates fall far short. In a Portuguese study, over 90% of cats received antiparasitics, but only 38% of owners dewormed quarterly for endoparasites and 28% dewormed quarterly for ectoparasites. Just 2% followed monthly deworming, while 26% used ectoparasiticides monthly [20].

The success of parasite control relies heavily on two key factors: owner willingness and ability to comply with the recommended treatment and veterinarians’ communication skills. To ensure better compliance, it is crucial to understand what influences owners’ decisions when selecting antiparasitic products for their cats and to consider these factors during client communication. While previous studies have identified barriers to compliance like dosage frequency, the complexity of using multiple products, the difficulty of application, and insufficient knowledge about available products, parasitic diseases, and their public health significance [18,21,22,23], there remains a need for a more systematic understanding of how cat owners prioritize and weigh different product attributes in their decision-making process.

To address this knowledge gap, our study employs a discrete choice experiment (DCE)—a methodological approach that has demonstrated robust results in both human and veterinary medicine [24]. A DCE offers several advantages over traditional preference assessment methods: it reduces measurement bias by simulating real-world decision-making scenarios, forces explicit trade-offs between attributes, and enables the quantification of relative importance for different factors. While DCEs have been successfully applied to various veterinary contexts and pet owner communication preferences regarding willingness to pay for recommended services, antimicrobials, and antiparasitic treatments for dogs [23,24,25,26,27], its application to preferences for feline antiparasitic treatments represents a novel opportunity to understand this specific aspect of feline healthcare. Since this preference process involves complex trade-offs, a method like a DCE that forces realistic choices is required to reveal true owner preferences.

To this end, we employed a multifaceted approach comprising a physical properties study (Phase 1), an ease-of-use study (Phase 2), and a DCE study (Phase 3) to identify cat owners’ preferences for feline antiparasitics and the key drivers influencing their preferences. By understanding these aspects, we aim to provide insights that can inform veterinarians to improve their communication strategies, ultimately increasing compliance rates and enhancing feline health and public health outcomes.

## 2. Materials and Methods

### 2.1. Study Overview

**Figure 1:**
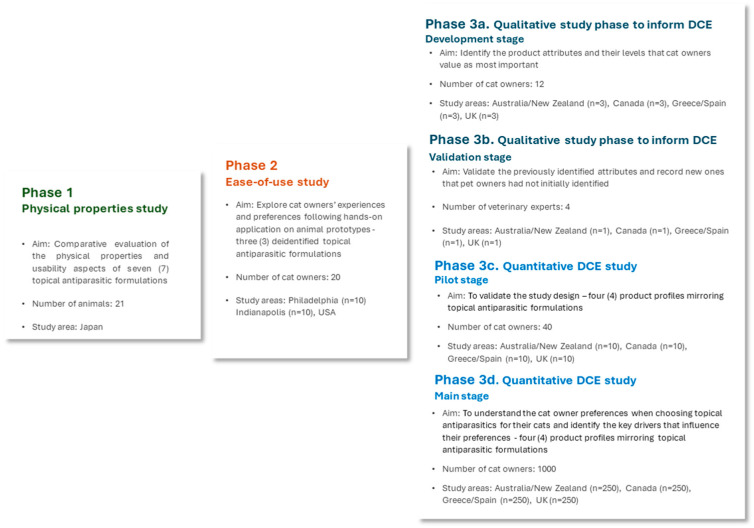
Study overview.

This study comprises three phases: (a) a physical properties study (Phase 1), (b) an ease-of-use study (Phase 2), and (c) a DCE study (Phase 3). An overview of the three study Phases is presented in Figure 1.

Phase 1, a physical properties study, comparatively evaluated the features and usability aspects of seven commercially available topical antiparasitic formulations for cats. Within Phase 2, an ease-of-use study was conducted, which involved pet owners evaluating the application experience of three deidentified topical antiparasitic formulations. Phase 3 comprised a qualitative study phase to inform the DCE, during which the product attributes most valued by cat owners were identified through cat owner interviews (development stage) and validated through consultations with veterinary experts (validation stage). The product attributes identified during the qualitative study phase informed the quantitative DCE survey, which included a pilot stage to gather preliminary insights into the study design and a main study focusing on pet owner preferences across product profiles mirroring four topical antiparasitic formulations in various countries.

### 2.2. Phase 1: Physical Properties Study

In this comparative study, product attributes, including the odor, stickiness, drying time properties, and viscosity, as well as certain usability aspects like the dosage, the volume of the solution, the administration process, and the container usability of seven spot-on antiparasitic formulations for cats were evaluated by the independent agency Shoku-kan-ken Inc. (561-21 Arakuchi-machi, Maebashi-shi, Gumma, Japan). The products assessed were designated as Treatments A, B, C, D, E, F and G corresponding to selamectin–sarolaner (Revolution/Stronghold Plus, Zoetis, Parsippany, NJ, USA), moxidectin–fluralaner (Bravecto Plus, Merck Animal Health, Madison, NJ, USA), moxidectin–imidacloprid (Advocate, Elanco, Greenfield, IN, USA), eprinomectin–esafoxolaner–praziquantel (Nexgard Combo, Boehringer Ingelheim, Ingelheim am Rhein, Germany), fipronil–(s)–Methoprene (Frontline Plus, Boehringer Ingelheim, Ingelheim am Rhein, Germany), moxidectin–imidacloprid, and moxidectin–imidacloprid (Advantage Multi, Elanco, Greenfield, IN, USA), respectively (Table 1).

#### 2.2.1. Enrolled Cats

A total of 21 clinically healthy male and female cats, aged 4 to 10 years, with no differences in type, fur, or skin condition, were enrolled in the study. The cats were randomly allocated into seven groups, each consisting of three cats. Body weight was measured immediately before product administration, ranging from 2.5 to 5.0 kg. Cats were housed individually in stainless-steel cages. They were fed a predetermined daily amount of food calculated based on body weight and administered once each morning. Additionally, the cats had ad libitum access to water via an automatic water dispenser.

#### 2.2.2. Study Design

For all the cats assigned to the seven groups, a single dose of the products under evaluation was administered by applying the solution to the skin along the midline of the neck, between the base of the skull and the shoulder blades.

#### 2.2.3. Assessment of Product Features

The researchers observed each cat’s general condition in terms of abnormalities immediately after the administration of each spot-on formulation. The product features of odor, stickiness, and drying time were scored using specific criteria (Table 2) at eight time points: 5, 10, 15, and 30 min, and 1, 4, 6, and 24 h.

The assessment of the products’ usability focused on the attributes of dosage, volume of solution, administration process, and container usability. The scoring system used was based on specific criteria summarized in Table 3.

The viscosity measurement was performed three times for each product using a tuning-fork vibration viscometer (SV-10, A&D Company, Limited, Tokyo, Japan).

### 2.3. Phase 2: Ease-of-Use Study

In the ease-of-use study, cat owners assessed their application experiences with deidentified products representing formulations combining selamectin–sarolaner, moxidectin–fluralaner, and eprinomectin–esafoxolaner–praziquantel.

#### 2.3.1. Participants and Inclusion Criteria

Potential participants were enrolled in the study by a recruiter guided by a screener, which was the basis of the study’s inclusion/exclusion criteria. Participants were introduced to the study’s nature and assured about confidentiality. They verified eligibility by sharing a picture of the current antiparasitic treatment used on their cat. Various demographic and qualifying questions were asked, including gender, age, cat ownership, veterinary visits, and product usage. Participants were also asked about their role in health decisions and their comfort handling products. A crucial aspect of screening was ensuring that participants would be comfortable discussing product attributes in detail.

#### 2.3.2. Evaluation of Cat Owners’ Application Experience

Τen dyadic interviews were conducted with 20 cat owners (ten from Philadelphia, Pennsylvania and ten from Indianapolis, IN, USA). Each dyad evaluated the ease of use of the application device for three blinded topical antiparasitic formulations.

The three products evaluated were labelled as Treatment A, B, or D, representing the topical formulations of selamectin–sarolaner, moxidectin–fluralaner, and eprinomectin–esafoxolaner–praziquantel, respectively. The moderator guided the discussions during the interviews using a comprehensive interview guide. Each dyadic interview session lasted approximately 60 min.

**Figure 2:**
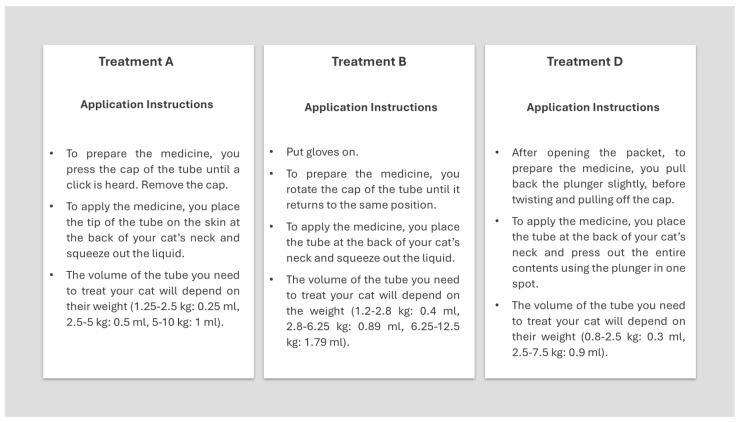
Example of the application instructions for the treatment formulations under evaluation.

During the interviews, participants applied each product to a plush cat toy (hereafter referred to as an “animal prototype”) to simulate the look and feel of a cat. Product identities remained concealed to ensure unbiased assessments. Blinded to the products, participants described their experiences applying products to animal prototypes. Each participant was provided gloves for each application and instructions on how to open each applicator and apply the product to the animal prototype (Figure 2).

### 2.4. Phase 3a: Qualitative Study Phase to Inform DCE—Development Stage

In the development stage of the qualitative study phase, 12 cat owners from Australia/New Zealand (n=3), Canada (n = 3), Greece/Spain (n = 3), and the UK (n = 3) who aligned with predefined screening criteria were recruited and interviewed. The interview included a mixture of structured open-ended and closed questions regarding preventative treatments for feline parasites, characteristics of an “ideal” preventative treatment, and usability drivers influencing treatment choice. During the interview, participants were presented with blinded treatment profiles and visual stimuli. They were asked for feedback on the content’s relevance, clarity, and completeness.

Participants were also asked to rank-order all the attributes based on their perceived importance (1: lowest level of importance to 7: highest level of importance) when choosing a topical preventative therapy for feline parasitic infection/infestation.

### 2.5. Phase 3b: Qualitative Study Phase to Inform DCE—Validation Stage

Four veterinary experts, including veterinarians, veterinary nurses/technicians, and pet retail specialists from Australia/New Zealand (n = 1), Canada (n = 1), Greece/Spain (n = 1), and the UK (n = 1) who aligned with predefined screening criteria were recruited and interviewed.

The interview included open-ended and closed questions to (a) identify who primarily influences the decisions and recommendations made by participants, as well as the motivations behind therapy recommendations; (b) capture the therapy options currently offered—or previously offered—for preventing parasitic infections in cats, including a breakdown of the most prescribed treatments; (c) explore the satisfaction levels of both prescribers and cat owners with current preventatives, focusing on ease of administration and usability in terms of formulations and applications; (d) examine how satisfaction influences cat owner behaviors related to ease of use and compliance, and how preventative therapy impacts the attitudes of cat owners and veterinarians towards feline behaviors and welfare; and (e) test product profiles and attribute descriptions for general comprehension, ensuring that all product profiles were blinded to cat owners.

The veterinary experts were also asked to rank-order all the attributes based on their perceived importance (1: lowest level of importance to 7: highest level of importance) when choosing a topical preventative therapy for feline parasitic infection/infestation. Participating veterinary experts were blinded to the products.

### 2.6. Phase 3c: Quantitative DCE Survey—Pilot Stage

A pilot study with 40 cat owners (10 from each market: Australia/New Zealand, Canada, Greece/Spain, and the UK) was conducted. The final survey tool was refined based on the pilot data.

### 2.7. Phase 3d: Quantitative DCE Survey—Main Stage

An international web-based survey was conducted with cat owners from Australia/New Zealand, Canada, Greece/Spain, and the UK, with a target sample size of *n* = 260 per country. The inclusion criteria were cat owners owning at least one cat and no more than four cats, with previous experience with topical preventative treatment for parasitic infections and infestations, aged ≥ 18 years, residents in one of the countries of interest, primary caretakers/decision-makers for their cat(s), and not employees in animal health or market research.

**Figure 3:**
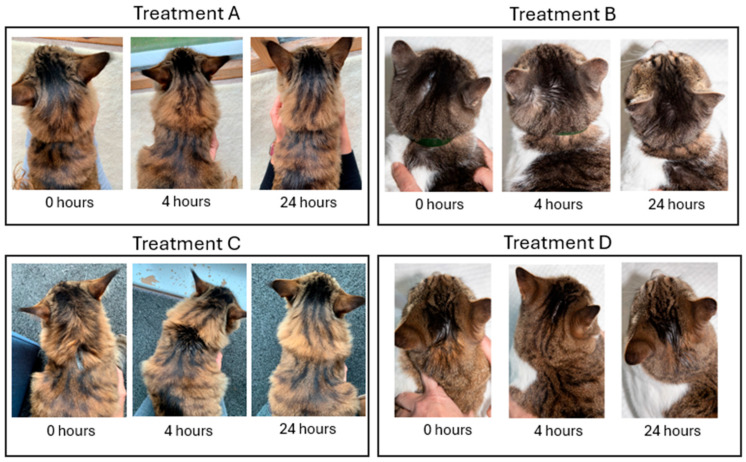
Photographs of cats following the application of the products corresponding to the treatment profiles under evaluation in different time intervals (0, 4, and 24 h).

The survey had an estimated completion time of 45 min. The initial section contained screening questions and an informed consent form. An introduction defined the set of attributes and their varying levels used in the study. They were provided with treatment profiles, each designed according to product labels that were accurate for each region at the time of the study. Each treatment profile was referred to anonymously (i.e., Treatment A, Treatment B) and mirrored a topical antiparasitic formulation: selamectin–sarolaner (Treatment A), moxidectin–fluralaner (Treatment B), moxidectin–imidacloprid (Treatment C), and eprinomectin–afoxolaner–praziquantel (Treatment D). Each profile was based on product labels accurate for the region and was accompanied by photographs of cats at 0, 4, and 24 h post-application (Figure 3).

The survey comprised closed-ranking or preference-based questions, covering demographics and experience with topical therapies. Participants rated the importance of different product attributes when choosing a topical preventative therapy for their cat using a seven-point scale, where one represented “not very important” and seven signified “very important”.

In the subsequent DCE, participants were presented with choice sets in which they had to choose between the following treatment profiles: Treatment A vs. Treatment B, Treatment A vs. Treatment C, and Treatment A vs. Treatment D. The presentation order of the choice sets and their visual presentation on the screen (left-hand vs. right-hand positions) were randomized.

### 2.8. Data Analysis

Descriptive statistics were employed to analyze the data collected from the physical properties study. For each product, mean endpoint total scores, mean score values for odor, stickiness, and drying time, and the mean viscosity values were calculated.

Qualitative data originating from the ease-of-use study, in the form of direct quotes from participants, was summarized using thematic analysis to identify patterns and themes.

Demographic data were analyzed using descriptive statistics, including means, medians, standard deviations, and confidence intervals. Preference data of respondent subgroups for the different treatment profiles were analyzed using the chi-squared test.

The relative importance of product attributes and their effects on treatment preferences were compared using regression analysis. Statistical significance for differences between subgroups was set at *p* < 0.05. All statistical analyses were conducted using R software (version 4.3.3).

## 3. Results

### 3.1. Phase 1: Physical Properties Study Results

**Figure 4:**
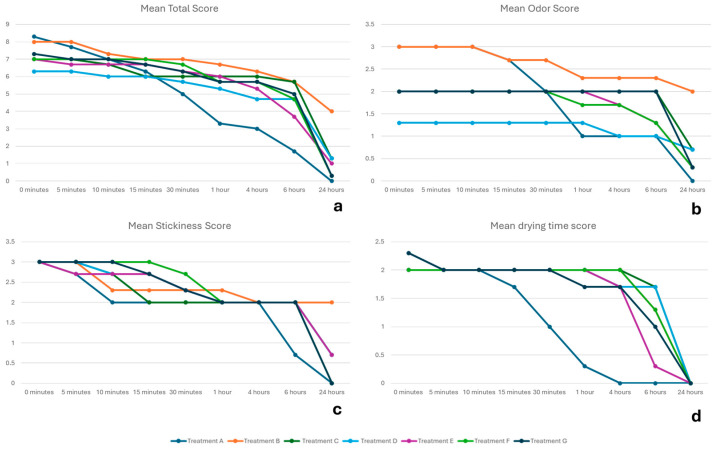
Changes over time in the mean scores for total endpoint (**a**), odor (**b**), stickiness (**c**), and drying time (**d**) assessments for the antiparasitic formulations under evaluation. A lower score indicated less odor, less stickiness, and quicker dry time. Treatment A: selamectin–sarolaner; Treatment B: moxidectin–fluralaner; Treatment C: moxidectin–imidacloprid; Treatment D: eprinomectin–afoxolaner–praziquantel; Treatment E: fipronil–(s)–methoprene; Treatment F: moxidectin–imidacloprid; Treatment G: moxidectin–imidacloprid.

The total score for Treatment A decreased as early as 5 min post-application, showing the lowest score among the comparative products by 30 min (Figure 4a). From one hour onward, Treatment A maintained the lowest total score, reaching a zero score in 24 h.

While Treatment D exhibited the lowest total score at the beginning of the study and showed a gradual decline over time, its total score consistently exceeded that of Treatment A from 30 min to 24 h post-application. At this time point, Treatment D showed the second highest total score among the tested products, while Treatment B had the highest total score, exceeding the comparative products (e.g., 4.0 for Treatment B versus 0.0 for Treatment A).

Upon examining each physical property individually, it was observed that Treatments A and B exhibited the highest odor scores at the onset of the study (Figure 4b). However, Treatment A demonstrated a decline in odor score over time, achieving the lowest score among all treatments at one-hour post-application and a complete absence of odor at 24 h post-application.

Regarding stickiness, Treatment A demonstrated the lowest score as early as 10 min post-application (Figure 4c). This was followed by a decline at 6 h post-application—a trend not observed in the comparative products. Only Treatments A, F, and G scored zero at 24 h post-application.

As for drying time, Treatment A showed the lowest score as early as 15 min post-application and maintained the lowest score at all subsequent time points (Figure 4d). Importantly, by 4 h post-application, Treatment A was completely dry (score 0.0)—a score that the other treatments achieved at 24 h.

Regarding the usability scores, all products evaluated received mean scores of zero, except Treatment G (0.7 vs. 0.0) (Table S1). This indicates that administration of all the products was easy, and the volume of the solution was suitable except for Treatment G, which was considered slightly large. The administration process according to the dosing regimen did not result in drug spillage, and the container usability was appropriate, allowing for precise administration by adjusting the dosage gradually.

**Figure 5:**
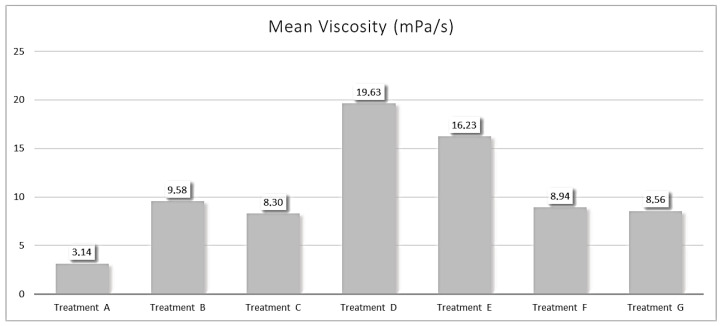
Mean viscosity measurements for the antiparasitic formulations under evaluation. The bars represent the mean viscosity (mPa/s) for each treatment. The data labels show the exact mean viscosity value for each treatment. Treatment A: selamectin–sarolaner; Treatment B: moxidectin–fluralaner; Treatment C: moxidectin–imidacloprid; Treatment D: eprinomectin–afoxolaner–praziquantel; Treatment E: fipronil–(s)–methoprene; Treatment F: moxidectin–imidacloprid; Treatment G: moxidectin–imidacloprid.

Finally, Treatment A showed the lowest mean viscosity value (3.14 mPa/s), followed by 8.30 mPa/s for Treatment C. Notably, Treatment D and E had the highest viscosity values of 19.6 mPa/s and 16.2 mPa/s, respectively (Figure 5 and Table S2).

### 3.2. Phase 2: Ease-of-Use Study Results

For Treatment A, participants’ application experience emphasized its seamless and efficient application process, characterized by rapid and hassle-free dispensing mechanisms. Consistently, participants reported a sense of control during the application, noting variances in the pressing and clicking mechanisms.

While Treatment B was positively associated with a user-friendly design and favorable product perception, it also raised concerns regarding the application efficiency and the requirement for gloves.

Treatment D presented an initial learning curve, rapid dispensing, and unexpected challenges during application.

### 3.3. Phase 3a and 3b: Qualitative Results to Inform DCE

Attributes identified by cat owners and veterinary experts during Phase 3a and Phase 3b informed those chosen for the DCE quantitative study (Table 4).

### 3.4. Phase 3c and 3d: Quantitative Results from DCE

#### 3.4.1. Cat Owner Demographics

When the pilot study was completed, a total of 1040 responses from cat owners across three continents were collected, corresponding to 250 responses from each country (Australia/New Zealand, Canada, Greece/Spain and the UK), and they were included in the final analysis together with the 40 responses collected during the pilot study. Preventative treatment profiles designed to mirror four topical antiparasitics—selamectin–sarolaner (Treatment A), moxidectin–fluralaner (Treatment B), moxidectin–imidacloprid (Treatment C), and eprinomectin–esafoxolaner–praziquantel (Treatment D)—were presented to the DCE study participants. Survey results were collated, cleaned, quality-checked, and analyzed.

The study population exhibited balanced representation across genders and coverage of all ages (Table 5). The average age of participants was 49.3 years (SD = 14.7). Most participants possessed an undergraduate degree (34.9%) and were engaged in full-time employment (57.4%).

#### 3.4.2. Cat Owner Experience with Parasites

Fleas were identified by 49% of the participants as the most important parasite for a topical preventative treatment to target. Intestinal parasites followed closely, being recognized by 30% of participants as the most important parasites (Table 6). This could be further explained by the fact that 50% and 17% of the surveyed participants reported that their cats had been previously infested with fleas and intestinal parasites, respectively. Additionally, ticks were deemed “important” parasites by 33% of respondents, and 15% reported previous infestations in their cats.

#### 3.4.3. The Perceived Importance of Product Attributes Among Cat Owners

Based on the importance ratings assigned by cat owners when selecting treatment options for their pets, 11 product attributes received ratings exceeding 5, indicating their importance on the seven-point scale (Table 7). Seven of these attributes are categorized under “Overall Ease of Use”. The remaining highly rated attributes pertain to “Negative Precautions Following Administration” and “Physical Properties” categories. Notably, the four attributes rated as being the most important to pet owners (≥6) were exclusively linked to “Overall Ease of Use”.

#### 3.4.4. Global Treatment Preferences Among Cat Owners

Across a global population of cat owners (N = 1040), Treatment A was statistically significantly preferred over Treatment B (54.7% vs. 45.3%, *p* = 0.01, Treatment C (61.7% vs. 38.3%, *p* = 0.01), and Treatment D (72.2% vs. 27.8%, *p* = 0.01) (Table S3).

When analyzing preferences based on cat owners’ countries of origin, Treatment A was favored over all other treatments, except for the case of Australia/New Zealand, where the difference was not statistically significant (*p* > 0.05).

#### 3.4.5. The Impact of Demographics on Cat Owner Preferences Across the Four Treatment Profiles

Demographic characteristics, such as gender, age, and insurance status, influenced participants’ treatment preferences.

A significantly higher percentage of male cat owners preferred Treatment A over Treatment B (56% vs. 44%, *p* < 0.01), Treatment C (60.2% vs. 39.8%, *p* < 0.01), and Treatment D (70.8% vs. 29.2%, *p* < 0.01) (Table S4). A similar pattern was observed among female cat owners, except in the comparison between Treatments A and B. Although more female participants preferred Treatment A, the difference was not statistically significant (53.5% vs. 46.5%, *p* > 0.05). A significantly higher percentage of female cat owners preferred Treatment A over Treatment C (62.8% vs. 37.2%, *p* < 0.01) and Treatment D (73.4% vs. 26.6%, *p* < 0.01) (Table S4).

Regarding age, a statistically significant preference for Treatment A over Treatment B was noted in participants aged 31–50 years (*p* < 0.05). Additionally, a statistically significant preference for Treatment A over Treatment C was recorded for all cat owners aged 31 years and older (*p* < 0.01). Finally, a statistically significant preference for Treatment A over Treatment D was observed across all age ranges (*p* < 0.01) (Table S4).

Lacking insurance positively influenced cat owners’ preference for Treatment A over Treatments C and D (*p* < 0.01) but not for Treatment B (*p* > 0.05). Conversely, having insurance was associated with a preference for Treatment A over Treatments B and D (*p* < 0.01) (Table S4).

#### 3.4.6. The Impact of the Perceived Product Attribute Importance on Cat Owner Preferences Across Four Treatment Profiles

The cat owners’ treatment preferences based on eight specific product attributes were used in this analysis. Cat owners were categorized into two groups based on their importance ratings: those who selected the maximum importance rating of seven (very important) and those who selected a rating of six or lower (not very important).

Among cat owners who regarded the product attributes as “very important”, a greater preference for Treatment A was observed compared to Treatment B for three out of eight attributes: (a) the ability to confirm successful administration of treatment (55% vs. 46%, *p* < 0.05), (b) drying time (63% vs. 37%, *p* < 0.01), and (c) the length of time before the cat can be touched following application (58% vs. 42%, *p* < 0.05) (Table S5). Notably, there were no instances in which cat owners preferred Treatment B over Treatment A.

Among cat owners who perceived the examined attributes as “not very important”, a significantly higher proportion preferred Treatment A over Treatment B across all assessed attributes (*p* < 0.05). The only exception was drying time, where although a greater proportion of cat owners favored Treatment A, no statistically significant difference was observed. Notably, there was no instance in which cat owners preferred Treatment B formulation over Treatment A.

Regarding the other two sets of treatment comparisons, among cat owners who classified the examined attributes as either “very important” or “not very important”, a statistically significant higher proportion (*p* < 0.05) preferred Treatment A over Treatment C and Treatment D across all analyzed attributes.

#### 3.4.7. The Relative Importance of Product Attributes and the Associated Effect on Treatment Preferences

Table 8, Table 9, Table 10 and Table 11 summarize the regression analysis results examining the preference for Treatment A over Treatment B, C, and D, considering the relative importance of product attributes for the participants. The analysis is based on 893 observations rated as 7 (very important). Each table includes only the significant results and predictors for treatment preferences. The statistically significant and non-significant regression analysis results are shown in Table S6. Regression analysis was also conducted within different age groups; however, no statistically significant models were found and are not presented herein.

The regression analysis identified several significant predictors influencing the preference for Treatment A over Treatment B (Table 8). Cat owners who rated the ability to confirm successful administration from the applicator device as very important were more likely to prefer Treatment A (β = 0.111, *p* < 0.05). Similarly, the importance placed on age restrictions of the cat also increased the preference for Treatment A (β = 0.120, *p* < 0.05). Conversely, concerns about the risk of treatment transfer to family members or furniture before drying reduced the preference for Treatment A (β = −0.110, *p* < 0.01).

For the comparison between Treatment A and Treatment C, significant predictors included the perceived length of time before the cat could be touched or rest on the furniture post-application (β = −0.126, *p* < 0.05) and the preparation required for the applicator device (β = −0.105, *p* < 0.05). Both factors decreased the likelihood of preferring Treatment A (Table 9).

In the comparison between Treatment A and Treatment D, the ease of use of the applicator device during the application step emerged as a significant predictor, favoring Treatment A (β = 0.081, *p* < 0.05) (Table 10). Additionally, the time before the cat could sit or sleep on furniture following application was a positive predictor (β = 0.104, *p* < 0.05). In contrast, the oiliness of the treatment formulation was a negative predictor (β = −0.133, *p* < 0.05).

While several other predictors were included in the analysis, they did not reach statistical significance at the conventional levels (Tables S6–S8).

The F statistic across the three models exploring the predictors that significantly influence preferences across the three comparisons (Treatment A vs. Treatment B, Treatment A vs. Treatment C and Treatment A vs. Treatment D) indicates varying levels of statistical significance (Table 11).

For the first model (Treatment A vs. Treatment B), an F statistic of 2.326 (*p* < 0.01) suggests that at least one predictor is significantly related to the preference for Treatment A. However, the model’s overall explanatory power is limited, as reflected by low R^2^ and adjusted R^2^ values and considerable variability indicated by the residual standard error. In contrast, the subsequent models (Treatment A vs. Treatment C and Treatment A vs. Treatment D) show lower F statistics, indicating that these models lack statistical significance and robustness.

## 4. Discussion

Our study provides comprehensive insights into cat owners’ preferences for antiparasitic treatments through a multifaceted approach: the evaluation of physical properties and usability aspects of seven topical antiparasitics (Phase 1), the evaluation of the application experience of deidentified products representing three antiparasitics by cat owners (Phase 2), and the international quantitative DCE study for cat owner preferences when choosing antiparasitics for their cats (Phase 3). This integrated methodology revealed that treatment selection is influenced by an intricate interplay of physical properties, application experience, usability aspects, and owner–pet dynamics, with implications for both veterinary practice and product development.

The physical properties study showed that topical antiparasitics for cats vary in odor, stickiness, and drying time. To this end, the selamectin–sarolaner formulation (Treatment A) showed the lowest odor score one hour after application, with a complete absence of odor at 24 h. This aspect is especially important for cats living indoors, where lingering odors can be unpleasant for the owners. Regarding stickiness, this formulation had the lowest score at 10 min post-application and exhibited no stickiness by 24 h. Non-stickiness is important for topical antiparasitics to avoid adherence to the cat’s fur or the owner’s clothing, which could reduce the drug’s efficacy. Additionally, the selamectin–sarolaner formulation exhibited high speed of drying, achieving complete dryness within four hours, a score reached by the other formulations at 24 h. Quick drying is essential for topical products because it allows the treated animals, especially those living indoors, to return to normal activities sooner and provide peace of mind to the cat owner. Finally, the selamectin–sarolaner formulation showed the lowest viscosity compared to the other formulations.

These differences can be attributed to the specific excipients used, particularly the choice of solvents [28]. Supporting our findings, the study by Chansiripornchai and Jantanawaranon (2020) emphasized the importance of stickiness and drying time as key usability parameters in cat spot-on parasiticides [29]. Their research demonstrated that formulations like selamectin and fluralaner presented reduced stickiness and faster drying times, achieving complete dryness within 24 h post-application [29]. Understanding these formulation characteristics can help veterinarians make informed recommendations based on the user experience and their specific needs, ultimately improving adherence to prescribed therapies.

Considering the usability aspects, that is, the product characteristics that enable patients and caregivers to effectively use pharmaceutical products in their everyday environments [30], we assessed the dosage, the solution volume, the administration process, and the container usability. This evaluation showed that the administration was straightforward, and the solution volumes were appropriate for all the products, except for the case of Treatment G (moxidectin–imidacloprid formulation for >4 kg to 8 kg). Moreover, the dosing regimen effectively minimized drug spillage, and the container design facilitated precise administration, allowing for gradual dosage adjustments. This finding underscores the overall user-friendliness of the formulations, which is crucial for ensuring adherence to treatment protocols. To this end, veterinarians should remember that the application experience associated with topical formulations is particularly important, as pet owners may administer these treatments themselves.

Although similar studies in veterinary medicine are lacking, studies on patients’ preferences in human medicine have repeatedly shown that the physical properties of drugs, like color, size, taste, smell, and shape, are important determinants of patient acceptance and preferences [30]. These findings are relevant in veterinary practice, as pet owners, who are involved in their pets’ health care, may have similar emotional responses to the physical attributes of antiparasitic treatments. For instance, whether choosing a product or not can be influenced by its odor, drying time, and stickiness.

This link between usability aspects and the cat owner’s preference for one formulation over another was further validated by the ease-of-use study, which included the hands-on application of blinded products representing three topical antiparasitics by the cat owners on animal prototypes. This study complemented the physical properties study and delved deeper into the application process and user experience. The participants reported that the product representing the selamectin–sarolaner formulation had a seamless application, characterized by rapid dispensing and a sense of control for the cat owner. They also reported a hassle-free experience, contrasting it with Treatment B, which, while user-friendly, raised concerns about application efficiency and the need for gloves. Likewise, Treatment D presented rapid dispensing but unexpected challenges during application. These findings highlight the importance of minimizing stress for the cat owner and their cats during product application.

The perceived importance of product attributes among cat owners is critical for further understanding their preferences. To this end, during the quantitative DCE study, participants rated the importance of the product attributes. Eleven product attributes were perceived as important based on the ratings assigned, indicating their role in treatment selection. Notably, seven attributes were related to overall ease of use, underscoring the importance of user-friendly designs. Attributes such as the ease of use of the applicator device, the spectrum of parasites covered, the familiarity with current treatment, and the ability to confirm successful administration were highly rated. These insights emphasize the need for veterinarians to consider product usability when recommending treatments. Attributes related to negative precautions following administration and physical properties also garnered attention. However, the highest-rated attributes were exclusively linked to overall ease of use, highlighting a clear preference among cat owners for products that simplify the treatment process.

The findings from the quantitative DCE study further showed a preference for the product profile mirroring the selamectin–sarolaner formulation among a global sample of 1040 cat owners. When data from all the countries were considered together, it was significantly favored over other formulations in all comparisons. Seemingly, when preferences were analyzed based on the countries of origin of the cat owners, it remained significantly favored over all other treatment profiles, except in Australia/New Zealand, where the difference was not statistically significant (*p* > 0.05).

Demographic characteristics such as gender, age, and insurance status influenced the cat owner’s preferences. Male cat owners preferred the product profile mirroring the selamectin–sarolaner formulation over all the other formulations. At the same time, female owners significantly favored the product profile mirroring selamectin–sarolaner formulation over Treatments C (mirroring moxidectin–imidacloprid formulation), and D (mirroring eprinomectin–afoxolaner–praziquantel formulation). Age also played a role, with a significant preference for the product profile mirroring the selamectin–sarolaner formulation over Treatment B (mirroring moxidectin–fluralaner formulation) among those aged 31–50 years and across all ages over Treatments C (*p* < 0.01) and D (*p* < 0.01). Insurance status positively influenced preferences for the product profile mirroring the selamectin–sarolaner formulation over C and D (*p* < 0.01) but not B (*p* > 0.05). These findings indicate that demographics play a significant role in cat owners’ treatment preferences, which could help veterinarians make informed recommendations in daily practice.

The analysis of cat owner treatment preferences, when considering the perceived importance of the product attributes, showed that those who rated the product attributes as “very important” showed a significant preference for the product profile mirroring the selamectin–sarolaner formulation over Treatment B for the following attributes: ability to confirm successful administration, drying time, and the length of time before the cat can be touched following application. Even among those who rated the attributes “not very important”, the product profile mirroring the selamectin–sarolaner formulation was preferred over Treatment B for all attributes assessed, except for drying time, where no significant difference was found. Overall, cat owners favored the product profile mirroring the selamectin–sarolaner formulation, with significant differences noted in multiple scenarios compared to Treatments B, C, and D.

Further analysis of the data obtained through the quantitative DCE study identified several key predictors influencing the preference for the product profile mirroring the selamectin–sarolaner formulation over the comparative treatment profiles. In brief, cat owners who prioritized the ability to confirm successful administration and age restrictions were more likely to prefer the product profile mirroring selamectin–sarolaner formulation. Conversely, increased concerns about treatment transfer to family members or furniture following application decreased this preference. In comparing the product profile mirroring selamectin–sarolaner formulation with Treatment C, factors such as the time before the cat could be touched post-application and the preparation required for the applicator device negatively impacted the likelihood of preferring the product profile mirroring the selamectin–sarolaner formulation. For the comparison of the product profile mirroring the selamectin–sarolaner formulation vs. Treatment D, ease of use during application and the time before the cat could sit on furniture following application positively predicted preference for the product profile mirroring selamectin–sarolaner formulation. In contrast, the oiliness of the formulation negatively affected it. These findings collectively suggest that cat owners prioritize safety, ease of use, and the overall experience of administering treatments.

However, the overall explanatory power of the model for the comparison between the product profile mirroring the selamectin–sarolaner formulation and Treatment B was limited, and the models comparing the product profile mirroring selamectin–sarolaner formulation to Treatment C and D exhibited a lack of robustness. This suggests that identifying explanatory predictors for variance in pet owners’ treatment preferences is complex and requires the consideration of several other factors to build robust prediction models and reach solid conclusions.

For instance, treatment cost could affect treatment preferences. However, this factor was not considered in our study, and its impact should be investigated in future research. In our study, this was a deliberate design choice to isolate preferences for intrinsic product features that are consistent across countries, as standardizing cost across diverse international markets would have introduced significant experimental complexity. Treatment cost is often presumed to be an important barrier to pet owners’ compliance, which could be indirectly related to treatment preferences. However, in a study conducted in Hong Kong, the cost of preventive products was one of the least frequent reasons for not using tick prevention, with other factors such as lifestyle and lack of knowledge being more prominent barriers [31]. In the same context, a study showed that 69% of cat owners were willing to spend any amount necessary for their cats’ health [23]. Another study showed that cat owners may be less willing to pay for the recommended care than dog owners unless they clearly understand the needs and benefits [23]. Thus, the preferences of pet owners may constitute a dynamic situation influenced by several other factors, including cultural differences, awareness of health risks, and available products and understanding of the treatment benefits and needs. These scenarios highlight that pet owner preference is a complex and dynamic situation that requires further investigation using methods, like DCEs, capable of investigating these competing factors.

Another limitation of this study was that the physical properties assessment, while comprehensive, was conducted under controlled laboratory conditions, and experienced researchers applied the treatments. These conditions may not fully reflect real-world application scenarios. Environmental factors such as temperature, humidity, and the variability in the cat owners’ application technique could influence the performance of these formulations in practice. In addition, while the quantitative DCE study provides valuable insights, it has inherent limitations. While our sample size of 1040 participants provided substantial statistical power, the self-selected nature of participation could have potentially led to overrepresenting more engaged and conscientious pet owners. As previously mentioned, the product labels were accurate for each country when the study was conducted, which may account for differences such as the recommended separation times before the cat could be touched after the product application. Finally, while our DCE used blinded product profiles, pre-existing familiarity with certain product characteristics or underlying active ingredients could still influence choices and should be explored in future research. Despite the above-mentioned limitations, this study provides valuable insights into pet owners’ preferences for spot-on antiparasitics in cats, supported by three distinct assessments: a physical properties study, an ease-of-use study, and a quantitative DCE study. Combining different methodologies enhances the validity of the key attributes that matter most to pet owners.

## 5. Conclusions

This study demonstrates that successful antiparasitic treatments must balance optimal physical properties with user-friendly application methods. The preference for the product profile mirroring the selamectin–sarolaner formulation suggests that formulations combining favorable physical characteristics with efficient application systems may achieve better owner preference and, potentially, improved treatment compliance. These findings provide valuable guidance for veterinary practitioners. While clinical efficacy remains fundamental, our results demonstrate that cat owners’ treatment preferences depend heavily on user- and animal-centric features. The importance of ease-of-use attributes, safety-related features, and physical properties suggests that veterinarians should adopt a holistic approach to treatment recommendations, considering both therapeutic efficacy and application characteristics.

## Figures and Tables

**Table 1:** Topical antiparasitic formulations evaluated in the physical properties study.

Treatment	Active Substances	Formulation	Dose/Cat	Number of Cats
A	Selamectin–sarolaner	≥2.5 kg to <5.0 kg	1 pipette	3
B	Moxidectin–fluralaner	>2.8 kg to 6.25 kg	1 pipette	3
C	Moxidectin–imidacloprid	≥1 kg to <4 kg	1 pipette	3
D	Eprinomectin–esafoxolaner–praziquantel	2.5 kg to <7.5 kg	1 pipette	3
E	Fipronil–(s)–methoprene	≥1 kg	1 pipette	3
F	Moxidectin–imidacloprid	≤4 kg	1 pipette	3
G	Moxidectin–imidacloprid	>4 kg to 8 kg	1 pipette	3

**Table 2:** Criteria and scoring system used for the assessment of the product features odor, stickiness, and drying time.

Product Features	Scoring System			
	0	1	2	3
Odor	No smell	Slight smell	Moderate smell	Strong smell
Stickiness	No stickiness	Slight stickiness on the skin surface	The fur also becomes sticky	The fur is adhered to the skin
Drying time	Dry	Almost dry	The drug is still present on the skin surface	The drug drips or almost drips

**Table 3:** Criteria and scoring system used for the assessment of the usability aspects.

Usability Attributes	Scoring System		
	0	1	2
Dosage	Easy	Difficult to administer	NA
Volume of the solution	Appropriate and manageable volume	Slightly large volume	Quite large volume
Administration	Can be administered according to the dosing regimen (no spill of the drug)	Difficult to administer the drug as the dosing regimen (e.g., drug spillage, unable to administer in one place)	NA
Usability of the container	The drug can be administered gradually by adjusting the dosage incrementally	Can be given normally	The drug solution overflows excessively

NA: Not applicable.

**Table 4:** The product attributes and their levels identified during the development and validation stages of the quantitative phase study and the average importance of each attribute as rated by cat owners and veterinary experts.

Attributes	Levels	Average Importance (x/7)	
		Cat Owner	Veterinary Expert
Overall ease of use	Length of protection time against parasites	6.9	6.5
	Ease of use during application	6.6	6
	Frequency of administration	6.1	6.25
	Preparation required for application	5.9	5
	Need for gloves during application	4.4	4
	Cat owner familiarity/satisfaction with current treatment	NA *	4.5
	The ability to confirm when the treatment has been successfully administered from the applicator device	NA *	5.75
	The spectrum/number of parasites covered by the preventative therapy	NA *	7
Negative precautions following administration	Length of time before the cat can touch/rest on the furniture	5.8	3.75
	Length of time before the cat can interact with people	5.4	4.75
	Length of time before the cat can interact with other pets	5.4	4
	Restrictions by age of the cat	NA *	4.75
	Restrictions relating to reproduction	NA *	4.75
	The risk that the treatment will be transferred from the cat onto family members or furniture, before drying	NA *	4.75
Physical properties	Drying time	4.9	4.75
	Risk of creating a mess	4.8	4.5
	The volume required for adequate protection	4.6	5.5
	Oiliness	3.6	5
	Appearance of treatment at site of administration following application	3.5	5
	The smell of a treatment formulation	N = 6 **	4.75

* Some attribute levels were added by veterinary experts during the validation stage based on the feedback from the initial cat owner interviews; these attributes do not have average importance ratings for the cat owners (NA). ** Where possible, the number of cat owners mentioning this topic (N) has been added to the table.

**Table 5:** Cat owner demographics.

Demographics	Number of Participants (n)	Percentage (%)
Gender		
Male	480	46.2
Female	557	53.6
Other	2	0.2
Not to say	1	0.1
Age		
18–30	120	11.5
31–40	202	19.4
41–50	231	22.2
51–60	216	20.8
61–70	184	17.7
>70	87	8.4
Qualifications		
Primary School	8	0.8
Secondary school	183	17.6
Diploma or vocational qualification	285	27.4
Undergraduate degree	363	34.9
Postgraduate degree	173	16.6
Doctorate	28	2.7
Employment status		
Unemployed	89	8.6
Student	21	2
Retired	188	18.1
Part-time worker	145	13.9
Full-time worker	597	57.4
Total (N)	1040	100

**Table 6:** The occurrence of previous parasitic infection/infestation and the importance of feline parasites as reported by cat owners.

	Previous Infection/ Infestation	Rating of Importance of the Feline Parasites				
		1	2	3	4	5
Fleas	50%	49%	27%	13%	6%	5%
Intestinal worms	17%	30%	24%	21%	10%	16%
Ticks	15%	17%	33%	27%	14%	9%
Ear mites	13%	3%	11%	25%	36%	25%
Lice	5%	1%	6%	14%	34%	45%
No infestation	36%					

1: most important; 2: very important, 3: moderately important, 4: slightly important, 5: least important.

**Table 7:** The importance of product attributes as rated by the cat owners participating in the quantitative DCE study.

Product Attribute Levels	Rating (Mean Value)	SD *
The length of time (number of weeks/months) that cat is protected against parasites following the application of the topical preventative treatment ^1^	6.3	0.9
Ease of use of applicator device during application step ^1^	6.2	0.99
The spectrum/number of parasites covered by the topical preventative therapy ^1^	6.1	1.1
Familiarity/satisfaction with current treatment ^1^	6.0	1.0
The ability to confirm when the treatment has been successfully administered from the applicator device ^1^	5.9	1.2
The frequency of administration ^1^	5.8	1.2
The preparation required for applicator device (e.g., collecting from storage, removing packaging, priming the device) ^1^	5.6	1.4
The drying time for topical preventative therapy ^3^	5.3	1.4
The volume of medication being applied ^3^	5.2	1.5
The length of time before the cat can be touched/stroked again following the application of the topical preventative treatment ^2^	5.1	1.6
The length of time before the cat can sit or sleep on furniture or bed following the application of the topical preventative treatment ^2^	5.1	1.7
The risk that the treatment will be transferred from the cat on to family members or furniture, before drying ^2^	4.9	1.8
Restrictions by age of cat (e.g., ability to use treatment in young cats aged ≥ 6 weeks) ^2^	4.7	1.8
The risk of creating a mess during the application of a topical preventative treatment ^3^	4.7	1.8
The length of time before cats in the same household are able to interact with one another/help groom one another again following administration ^2^	4.6	2.0
The smell of a treatment formulation ^3^	4.5	1.9
The oiliness of a treatment formulation ^3^	4.5	1.7
The appearance of the hair/coat at the site of application following treatment ^3^	4.3	1.9
The need for gloves during application ^1^	4.2	1.9
Restrictions relating to reproduction (e.g., ability to use treatment in pregnant or nursing cats) ^2^	4.0	2.2

* SD: Standard Deviation. ^1^ Product attributes that are included in the broader category of “Overall ease of use”. ^2^ Product attributes that are included in the broader category of “Negative precautions following administration”. ^3^ Product attributes that are included in the broader category of “Physical properties”.

**Table 8:** Significant predictors of preference for Treatment A over Treatment B.

Predictor	Coefficient (β)	Standard Error	Significance Level
Ability to confirm successful administration of treatment	0.111	0.043	*p* < 0.05
Restrictions by age of the cat	0.120	0.054	*p* < 0.05
The risk that the treatment will be transferred from the cat onto family members or furniture, before drying	−0.110	0.051	*p* < 0.01

**Table 9:** Significant predictors of preference for Treatment A over Treatment C.

Predictor	Coefficient (β)	Standard Error	Significance Level
Length of time before the cat can touch/rest on the furniture	−0.126	0.054	*p* < 0.05
Preparation required for application	−0.105	0.046	*p* < 0.05

**Table 10:** Significant predictors of preference for Treatment A over Treatment D.

Predictor	Coefficient (β)	Standard Error	Significance Level
Ease of use of applicator device	0.081	0.040	*p* < 0.05
Oiliness	−0.133	0.061	*p* < 0.05
Length of time before the cat can touch/rest on the furniture	0.104	0.051	*p* < 0.05

**Table 11:** Model metrics summary.

Comparison	R^2^	Adjusted R^2^	Residual Std. Error	F Statistic (*p*-Value)
Treatment A vs. Treatment B	0.053	0.030	0.493	2.326 (*p* < 0.01)
Treatment A vs. Treatment C	0.036	0.013	0.483	1.541 (*p* < 0.1)
Treatment A vs. Treatment D	0.025	0.002	0.454	1.078 (*p* > 0.1)

## Data Availability

The original contributions presented in this study are included in the article/Supplementary Material. Further inquiries can be directed to the corresponding author(s).

## Supplementary Materials

The following supporting information can be downloaded at: https://www.mdpi.com/article/10.3390/ani15131985/s1, Table S1: Usability scores for the seven topical antiparasitics evaluated; Table S2: Viscosity measurement results for the seven topical antiparasitics evaluated; Table S3: Treatment preferences among cat owners globally and per country; Table S4: Pet owner treatment preferences according to demographics; Table S5: The impact of the perceived product attribute importance on pet owner preferences when choosing among the different treatment profiles; Table S6: Regression analysis. Predictors of preference for Treatment A vs. Treatment B; Table S7: Regression analysis. Predictors of preference for Treatment A vs. Treatment C; Table S8: Regression analysis. Predictors of preference for Treatment A vs. Treatment D.
