# Supplementary material for: A Discrete Choice Experiment to Assess Cat Owners’ Preferences for Topical Antiparasitics and the Comparative Ease of Use of a Combined Selamectin and Sarolaner Formulation: An International Survey
Source: Animals (Basel). 2025 Jul 6;15(13):1985. doi: 10.3390/ani15131985 (PMC12248694; doi:10.3390/ani15131985)
Supplement: Supplementary file 1 [file animals-15-01985-s001.zip › animals-3615344-supplementary.pdf]

Supplementary Materials

Table S1. Usability scores for the seven topical antiparasitics evaluated

Treatment	Number of cats	Dosage	Volume of the solution	Administ ration	Usability of the container	Total Score	Mean	SD*
A	7	0	0	0	0	0		
	8	0	0	0	0	0	0	0
	9	0	0	0	0	0		
B	16	0	0	0	0	0		
	17	0	0	0	0	0	0	0
	18	0	0	0	0	0		
C	13	0	0	0	0	0		
	14	0	0	0	0	0	0	0
	15	0	0	0	0	0		
D	19	0	0	0	0	0		
	20	0	0	0	0	0	0	0
	21	0	0	0	0	0		
E	10	0	0	0	0	0		
	11	0	0	0	0	0	0	0
	12	0	0	0	0	0		
F	1	0	0	0	0	0		
	2	0	0	0	0	0	0	0
	3	0	0	0	0	0		
G	4	0	1	0	0	1		
	5	0	1	0	0	1	0.67	0.58
	6	0	0	0	0	0		

*SD: Standard Deviation

Table S2. Viscosity measurement results for the seven topical antiparasitics evaluated

Treatment	Measured value (mPa/s) (mPa.s)	Viscosity* (mPa/s) (mPa.s)	SD**
A	3.13		
	3.14	3.14	0.0152
	3.16		
B	9.63		
	9.57	9.58	0.0416
	9.55		
C	8.31		
	8.27	8.30	0.0230
	8.31		
D	19.6		
	19.6	19.6	0.0577
	19.7		
E	16.3		
	16.2	16.2	0.0577
	16.2		

F	8.94<br>8.94<br>8.93	8.94	0.0057
G	8.58<br>8.54<br>8.56	8.56	0.0200

* Mean of three measurement values
**SD: Standard Deviation

Table S3. Treatment preferences among cat owners globally and per country

Country	Treatment	N	Proportion (%)	Confidence Interval (95% CI)	p-value
All countries	A	569	54.7	0.5471 ± 0.0302	0.0023
	B	471	45.3	0.4529 ± 0.0302	
	A	642	61.7	0.6173 ± 0.0295	0.0000
	C	398	38.3	0.3827 ± 0.0295	
	A	751	72.2	0.7221 ± 0.0272	0.0000
	D	289	27.8	0.2779 ± 0.0272	
UK	A	147	56.5	0.5654 ± 0.0602	0.0349
	B	113	43.5	0.4346 ± 0.0602	
	A	166	63.8	0.6385 ± 0.0584	0.0000
	C	94	36.2	0.3615 ± 0.0584	
	A	186	71.5	0.7154 ± 0.0548	0.0000
	D	74	28.5	0.2846 ± 0.0548	
Canada	A	160	61.5	0.6154 ± 0.0591	0.0002
	B	100	38.5	0.3846 ± 0.0591	
	A	137	52.7	0.5269 ± 0.0606	0.3852
	C	123	47.3	0.4731 ± 0.0606	
	A	198	76.1	0.7615 ± 0.0518	0.0000
	D	62	23.9	0.2385 ± 0.0518	
Australia and New Zealand	A	118	45.4	0.4538 ± 0.0605	0.1366
	B	142	54.6	0.5462 ± 0.0605	
	A	182	70.0	0.7000 ± 0.0557	0.0000
	C	78	30.0	0.3000 ± 0.0557	
	A	172	66.2	0.6615 ± 0.0575	0.0000
	D	88	33.6	0.3385 ± 0.0575	
Spain and Greece	A	144	55.4	0.5538 ± 0.0604	0.0824
	B	116	44.6	0.4462 ± 0.0604	
	A	157	60.4	0.6038 ± 0.0594	0.0008
	C	103	39.6	0.3962 ± 0.0594	
	A	195	75.0	0.7500 ± 0.0526	0.0000
	D	65	25.0	0.2500 ± 0.0526	

Significance levels (chi-square test): * $p < 0.05$, ** $p < 0.01$

Table S4. Pet owner treatment preferences according to demographics

Demographics	Treatment	N	Proportion (%)	Confidence Interval (95% CI)	p-value
Male	A	269	56.0	0.5604 ± 0.0444	0.0081
	B	211	44.0	0.4396 ± 0.0444	
	A	289	60.2	0.6021 ± 0.0437	0.0000
	C	191	39.8	0.3979 ± 0.0437	
	A	340	70.8	0.7083 ± 0.0406	0.0000
	D	140	29.2	0.2917 ± 0.0406	
Female	A	298	53.5	0.535 ± 0.04142	0.0984
	B	259	46.5	0.465 ± 0.04142	
	A	350	62.8	0.6284 ± 0.0401	0.0000
	C	207	37.2	0.3716 ± 0.0401	
	A	409	73.4	0.7343 ± 0.0366	0.0000
	D	148	26.6	0.2657 ± 0.0366	
18-30 years old	A	68	56.7	0.5667 ± 0.0886	0.1441
	B	52	43.3	0.4333 ± 0.0886	
	A	62	51.7	0.5167 ± 0.0894	0.7150
	C	58	48.3	0.4833 ± 0.0894	
	A	82	68.3	0.6833 ± 0.0832	0.0001
	D	38	31.7	0.3167 ± 0.0832	
31-40 years old	A	117	57.9	0.5792 ± 0.0680	0.0244
	B	85	42.0	0.4208 ± 0.0680	
	A	122	60.4	0.604 ± 0.06744	0.0031
	C	80	39.6	0.396 ± 0.06744	
	A	141	69.8	0.698 ± 0.06331	0.0000
	D	61	30.2	0.302 ± 0.06331	
41-50 years old	A	138	59.7	0.5974 ± 0.0632	0.0031
	B	93	40.3	0.4026 ± 0.0632	
	A	141	61.0	0.6104 ± 0.0628	0.0008
	C	90	39.0	0.3896 ± 0.0628	
	A	181	78.4	0.7835 ± 0.0531	0.0000
	D	50	21.7	0.2165 ± 0.0531	
51-60 years old	A	112	51.9	0.5185 ± 0.0666	0.5862
	B	104	48.2	0.4815 ± 0.0666	
	A	130	60.2	0.6019 ± 0.0652	0.0028
	C	86	39.8	0.3981 ± 0.0652	
	A	155	71.8	0.7176 ± 0.0600	0.0000
	D	61	28.2	0.2824 ± 0.0600	
61-70 years old	A	87	47.3	0.4728 ± 0.0721	0.4610
	B	97	52.7	0.5272 ± 0.0721	
	A	127	69.0	0.6902 ± 0.0668	0.0000
	C	57	31.0	0.3098 ± 0.0668	
	A	130	70.7	0.7065 ± 0.0657	0.0000
	D	54	29.4	0.2935 ± 0.0657	

71+ years old	A	47	54.0	0.5402 ± 0.1047	0.4530
	B	40	46.0	0.4598 ± 0.1047	
	A	60	69.0	0.6897 ± 0.0972	0.0004
	C	27	31.0	0.3103 ± 0.0972	
	A	62	71.3	0.7126 ± 0.0950	0.0001
	D	25	28.7	0.2874 ± 0.0950	
Not insured	A	365	53.6	0.5360 ± 0.0374	0.0604
	B	316	46.4	0.464 ± 0.03746	
	A	454	66.7	0.6667 ± 0.0354	0.0000
	C	227	33.3	0.3333 ± 0.0354	
	A	504	74.0	0.7401 ± 0.0329	0.0000
	D	177	26.0	0.2599 ± 0.0329	
Insured	A	204	56.8	0.5682 ± 0.0512	0.0097
	B	155	43.2	0.4318 ± 0.0512	
	A	188	52.4	0.5237 ± 0.0516	0.3696
	C	171	47.6	0.4763 ± 0.0516	
	A	247	68.8	0.6880 ± 0.0479	0.0000
	D	112	31.2	0.3120 ± 0.0479	

* Statistical significance (p<0.05)
**Statistical significance (p<0.01)

Table S5. The impact of the perceived product attribute importance on pet owner preferences when choosing among the different treatment profiles

**Product attribute: rating**	**Treatment**	**N**	**Proportion (%)**	**Confidence Intervals (95% CI)**	**p-value**
Duration of protection: ≤6	A	296	58.3	0.5827 ± 0.0428	0.0002
	B	212	41.7	0.4173 ± 0.0428	
	A	313	61.6	0.6161 ± 0.0422	0.0000
	C	195	38.4	0.3839 ± 0.0422	
	A	368	72.4	0.7244 ± 0.0388	0.0000
	D	140	27.6	0.2756 ± 0.0388	
Duration of protection: 7	A	273	51.3	0.5132 ± 0.0424	0.5439
	B	259	48.7	0.4868 ± 0.0424	
	A	329	61.8	0.6184 ± 0.0412	0.0000
	C	203	38.2	0.3816 ± 0.0412	
	A	383	72.0	0.7199 ± 0.0381	0.0000
	D	149	28.0	0.2801 ± 0.0381	
Ease of use of applicator device: ≤6	A	321	56.9	0.5691 ± 0.0408	0.0010
	B	243	43.0	0.4309 ± 0.0408	
	A	340	60.3	0.6028 ± 0.0403	0.0000
	C	224	39.7	0.3972 ± 0.0403	
	A	396	70.2	0.7021 ± 0.0377	0.0000
	D	168	29.8	0.2979 ± 0.0377	
	A	248	52.1	0.5210 ± 0.0448	0.3593

Ease of use of applicator device: 7	B	228	47.9	0.4790 ± 0.0448	
	A	302	63.5	0.6345 ± 0.0432	0.0000
	C	174	36.5	0.3655 ± 0.0432	
	A	355	74.6	0.7458 ± 0.0391	0.0000
	D	121	25.4	0.2542 ± 0.0391	
Spectrum/ number of parasites covered: ≤6	A	306	55.0	0.5494 ± 0.0413	0.0198
	B	251	45.0	0.4506 ± 0.0413	
	A	333	59.8	0.5978 ± 0.0407	0.0000
	C	224	40.2	0.4022 ± 0.0407	
	A	410	73.6	0.7361 ± 0.0366	0.0000
	D	147	26.4	0.2639 ± 0.0366	
Spectrum/ number of parasites covered: 7	A	263	54.5	0.5445 ± 0.0444	0.0504
	B	220	45.6	0.4555 ± 0.0444	
	A	309	64.0	0.6398 ± 0.0428	0.0000
	C	174	36.0	0.3602 ± 0.0428	
	A	341	70.6	0.7060 ± 0.0406	0.0000
	D	142	29.4	0.2940 ± 0.0406	
Ability to confirm successful administration of treatment: ≤6	A	354	54.4	0.5438 ± 0.0382	0.0255
	B	297	45.6	0.4562 ± 0.0382	
	A	391	60.0	0.6006 ± 0.0376	0.0000
	C	260	40.0	0.3994 ± 0.0376	
	A	465	71.4	0.7143 ± 0.0347	0.0000
	D	186	28.6	0.2857 ± 0.0347	
Ability to confirm successful administration of treatment: 7	A	215	55.3	0.5527 ± 0.0494	0.0376
	B	174	44.7	0.4473 ± 0.0494	
	A	251	64.5	0.6452 ± 0.0475	0.0000
	C	138	35.5	0.3548 ± 0.0475	
	A	286	73.5	0.7352 ± 0.0438	0.0000
	D	103	26.5	0.2648 ± 0.0438	
Preparation required for applicator device: ≤6	A	413	54.4	0.5441 ± 0.0354	0.0150
	B	346	45.6	0.4559 ± 0.0354	
	A	481	63.4	0.6337 ± 0.0342	0.0000
	C	278	36.6	0.3663 ± 0.0342	
	A	550	72.5	0.7246 ± 0.0317	0.0000
	D	209	27.5	0.2754 ± 0.0317	
Preparation required for applicator device: 7	A	156	55.5	0.5552 ± 0.0581	0.0644
	B	125	44.5	0.4448 ± 0.0581	
	A	161	57.3	0.5730 ± 0.0578	0.0145
	C	120	42.7	0.4270 ± 0.0578	
	A	201	71.5	0.7153 ± 0.0527	0.0000
	D	80	28.5	0.2847 ± 0.0527	
Drying time: ≤6	A	426	52.5	0.5246 ± 0.0343	0.1604
	B	386	47.5	0.4754 ± 0.0343	
	A	507	62.4	0.6244 ± 0.0333	0.0000
	C	305	37.6	0.3756 ± 0.0333	

	A	585	72.0	0.7204 ± 0.0308	0.0000
	D	227	28.0	0.2796 ± 0.0308	
Drying time: 7	A	143	62.7	0.6272 ± 0.0627	0.0001
	B	85	37.3	0.3728 ± 0.0627	
	A	135	59.2	0.5921 ± 0.0637	0.0054
	C	93	40.8	0.4079 ± 0.0637	
	A	166	72.8	0.7281 ± 0.0577	0.0000
	D	62	27.2	0.2719 ± 0.0577	
Volume of medication to be applied: ≤6	A	442	54.7	0.5470 ± 0.0343	0.0075
	B	366	45.3	0.4530 ± 0.0343	
	A	498	61.6	0.6163 ± 0.0335	0.0000
	C	310	38.4	0.3837 ± 0.0335	
	A	583	72.2	0.7215 ± 0.0309	0.0000
	D	225	27.9	0.2785 ± 0.0309	
Volume of medication to be applied: 7	A	127	54.7	0.5474 ± 0.0640	0.1486
	B	105	45.3	0.4526 ± 0.0640	
	A	144	62.0	0.6207 ± 0.0624	0.000
	C	88	37.9	0.3793 ± 0.0624	
	A	168	72.4	0.7241 ± 0.0575	0.0000
	D	64	27.6	0.2759 ± 0.0575	
Length of time before cat can be touched: ≤6	A	434	53.8	0.5378 ± 0.0344	0.0318
	B	373	46.2	0.4622 ± 0.0344	
	A	497	61.6	0.6159 ± 0.0335	0.0000
	C	310	38.4	0.3841 ± 0.0335	
	A	579	71.8	0.7175 ± 0.0310	0.0000
	D	228	28.3	0.2825 ± 0.0310	
Length of time before cat can be touched: 7	A	135	57.9	0.5794 ± 0.0633	0.0154
	B	98	42.1	0.4206 ± 0.0633	
	A	145	62.2	0.6223 ± 0.0622	0.0002
	C	88	37.8	0.3777 ± 0.0622	
	A	172	73.8	0.7382 ± 0.0564	0.0000
	D	61	26.2	0.2618 ± 0.0564	

Significance levels (chi-square test): * $p < 0.05$, ** $p < 0.01$.

Table S6. Regression analysis. Predictors of preference for Treatment A vs. Treatment B

Predictor	Coefficient (β)	Standard Error	Significance Level
Experience with Previous Therapy	-0.055	0.039	
Ease of use of applicator device	-0.076	0.043	p < 0.1
Pet owner familiarity/ satisfaction with current treatment	-0.023	0.042	
Ability to confirm successful administration of treatment	0.111	0.043	p < 0.05
Need for Gloves	0.019	0.054	

Drying time	0.087 *	0.051	$p < 0.1$
Frequency of administration	-0.067	0.046	
The spectrum/number of parasites covered	0.051	0.039	
Length of protection time against parasites	-0.061	0.041	
Restrictions by age of the cat	0.120	0.054	$p < 0.05$
Restrictions Relating to Reproduction	-0.002	0.055	
Oiliness	0.052	0.066	
Volume required for adequate protection	-0.048	0.053	
Risk of creating a mess	0.096	0.053	$p < 0.1$
The risk that the treatment will be transferred from the cat onto family members or furniture, before drying	-0.110	0.051	$p < 0.01$
The smell of a treatment formulation	0.066	0.056	
Length of time before the cat can interact with people	-0.010	0.055	
Length of time before the cat can touch/rest on the furniture	-0.005	0.055	
Appearance of treatment at site of administration following application	0.062	0.066	
Preparation required for application	0.046	0.047	
Length of time before feline can interact with other pets	-0.027	0.051	
Constant	0.501	0.027	$p < 0.01$
F Statistic (df = 21; 871)	2.326		$p < 0.01$

$R^2 = 0.053$
Adjusted $R^2 = 0.030$
Residual Std. Error (df = 871) = 0.493

Table S7. Regression analysis. Predictors of preference for Treatment A vs. Treatment C

**Predictor**	**Coefficient (β)**	**Standard Error**	**Significance Level**
Experience with Previous Therapy	0.048	0.038	
Ease of use of applicator device	0.050	0.042	
Pet owner familiarity/ satisfaction with current treatment	0.057	0.041	
Ability to confirm successful administration of treatment	0.043	0.042	
Need for Gloves	-0.100	0.053	$p < 0.1$

Drying time	-0.006	0.050	
Frequency of administration	-0.044	0.045	
The spectrum/number of parasites covered	0.017	0.038	
Length of protection time against parasites	-0.022	0.040	
Restrictions by age of the cat	0.043	0.053	
Restrictions Relating to Reproduction	-0.054	0.054	
Oiliness	-0.056	0.064	
Volume required for adequate protection	0.045	0.052	
Risk of creating a mess	-0.071	0.052	
The risk that the treatment will be transferred from the cat onto family members or furniture, before drying	0.046	0.050	
The smell of a treatment formulation	-0.038	0.055	
Length of time before the cat can interact with people	0.072	0.054	
Length of time before the cat can touch/rest on the furniture	-0.126	0.054	$p < 0.05$
Appearance of treatment at site of administration following application	0.104	0.065	
Preparation required for application	-0.105	0.046	$p < 0.05$
Length of time before feline can interact with other pets	0.043	0.050	
Constant	0.601	0.027	$p < 0.01$
F Statistic (df = 21; 871)	1.541 *		$p < 0.1$

$R^2 = 0.036$
Adjusted $R^2 = 0.013$
Residual Std. Error (df = 871) = 0.483

Table S8. Regression analysis. Predictors of preference for Treatment A vs. Treatment D

**Experience with Previous Therapy**	**Coefficient (β)**	**Standard Error**	**Significance Level**
Ease of use of applicator device	-0.010	0.036	
Pet owner familiarity/ satisfaction with current treatment	0.081	0.040	$p < 0.05$
Ability to confirm successful administration of treatment	0.007	0.039	
Need for Gloves	0.016	0.040	
Drying time	-0.058	0.050	

Frequency of administration	0.008	0.047	
The spectrum/number of parasites covered	-0.036	0.042	
Length of protection time against parasites	-0.061	0.036	$p < 0.1$
Restrictions by age of the cat	-0.014	0.038	
Restrictions Relating to Reproduction	0.032	0.050	
Oiliness	0.006	0.051	
Volume required for adequate protection	-0.133	0.061	$p < 0.05$
Risk of creating a mess	0.014	0.048	
The risk that the treatment will be transferred from the cat onto family members or furniture, before drying	0.026	0.048	
The smell of a treatment formulation	-0.041	0.047	
Length of time before the cat can interact with people	0.076	0.051	
Length of time before the cat can touch/rest on the furniture	-0.023	0.051	
Appearance of treatment at site of administration following application	0.104	0.051	$p < 0.05$
Preparation required for application	-0.010	0.061	
Length of time before feline can interact with other pets	-0.045	0.043	
Constant	-0.003	0.047	
Experience with Previous Therapy	0.713	0.025	$p < 0.01$
F Statistic (df = 21; 871)	1.078		

$R^2 = 0.025$
Adjusted $R^2 = 0.002$
Residual Std. Error (df = 871) = 0.454
